# Downregulation of Deiodinase 3 is the earliest event in photoperiodic and photorefractory activation of the gonadotropic axis in seasonal hamsters

**DOI:** 10.1038/s41598-017-17920-y

**Published:** 2017-12-18

**Authors:** Sébastien Milesi, Valérie Simonneaux, Paul Klosen

**Affiliations:** Institute of Cellular and Integrative Neuroscience, CNRS, University of Strasbourg, 67084 Strasbourg, Cedex France

## Abstract

In seasonal rodents, reproduction is activated by a long photoperiod. Furthermore, maintaining an inhibitory short photoperiod for over 20 weeks triggers a spontaneous reactivation of the gonadotropic axis called photorefractoriness. Photoactivation is proposed to involve melatonin, hypothalamic thyroid hormones (TH) and (Arg) (Phe)-amide peptides. The mechanisms involved in photorefractoriness are so far unknown. We analyzed the dynamic changes in long photoperiod- and photorefractory-induced activation of reproduction in both Syrian and Djungarian hamsters to validate the current model of photoactivation and to uncover the mechanisms involved in photorefractoriness. We detected a conserved early inhibition of expression of the TH catabolizing enzyme deiodinase 3 (Dio3) in tanycytes, associated with a late decrease of the TH transporter MCT8. This suggests that an early peak of hypothalamic TH may be involved in both photoinduced and photorefractory reactivation. In photoactivation, Dio3 downregulation is followed by an upregulation of Dio2, which is not observed in photorefraction. The upregulation of (Arg) (Phe)-amides occurs several weeks after the initial Dio3 inhibition. In conclusion, we uncovered a so far unreported early inhibition of Dio3. This early downregulation of Dio3 is reinforced by an upregulation of Dio2 in photoactivated hamsters. In photorefractoriness, the Dio3 downregulation might be sufficient to reactivate the gonadotropic axis.

## Introduction

The earth’s rotation around the sun leads to annual changes in the duration of the daily light phase (called photoperiod), temperature and food resources. Organisms anticipate and adapt their physiology to these seasonal changes to optimize species survival. Rodents such as the Syrian (*Mesocricetus auratus*) or the Djungarian (*Phodopus sungorus)* hamster synchronize their metabolic and reproductive activities with the seasons. In mammals, the major seasonal synchronizer is the annual change in photoperiod length to which species respond differently according to their reproductive physiology. Hamsters are thus called long-day breeders because they are activated by a long photoperiod (LP), while a short photoperiod (SP) inhibits their reproduction. Interestingly, if hamsters are maintained for a prolonged period (over 20 weeks) in SP, their reproductive axis escapes the inhibitory action of the SP and endogenously reactivates. The mechanisms underlying this endogenous reactivation called photorefractoriness are so far unknown.

Early experiments have demonstrated that photoperiod is translated into circulating melatonin by the pineal gland. This nocturnal production of melatonin depends on the length of the night^1,2^. During the last decade, a number of genes have been shown to display melatonin-dependent photoperiodic variations leading to the elaboration of a neuroendocrine pathway through which melatonin is supposed to control reproductive activity. In this pathway, the large SP melatonin peak inhibits thyroid stimulating hormone (TSH) production in the *pars tuberalis* while the short LP melatonin peak relieves this inhibition^3,4^. This TSH is thought to bind to its receptor located on tanycytes, specialized glial cells lining the bottom of the 3^rd^ ventricle to increase deiodinase 2 (Dio2) and inhibit deiodinase 3 (Dio3) expression^5,6^. As Dio2 converts thyroxine (T4) to its active metabolite triiodothyronine (T3) and Dio3 catabolizes both T4 and T3 to inactive metabolites, TSH action on tanycytes leads to a local increase in T3 in the mediobasal hypothalamus (MBH)^7,8^. Tanycytes also express the blood to brain thyroid hormone transporter MCT8 known to be down regulated by photoperiod^9^. T3 is further proposed to increase the synthesis of two hypothalamic neuropeptides, Kisspeptin (KP) and RFamide related peptide-3 (RFRP-3), both well established regulators of GnRH neuron activity (^8,10,11^ see^12^ for review). This putative TSH-Dio2/Dio3/MCT8-RFRP/Kp pathway involved in the melatonin-driven control of reproductive activity has been tested by central infusion of TSH or T3, both of which are able to reactivate the HPG axis of photoinhibited animals^6–8,13,14^. However, these studies cannot exclude that these treatments might simply override the photoperiodic inhibition of the gonadotropic axis, and that a reactivation by photoperiod involves additional mechanisms, particularly in the early phases.

Thus we decided to analyze the dynamic changes along the whole putative neuroendocrine pathway in the course of a photoperiodic activation in order to uncover the sequential changes of this pathway. Furthermore, we analyzed the temporal changes of this neuroendocrine pathway during the endogenous photorefractory reactivation in order to compare the mechanisms involved in both the melatonin dependent and independent regulations of the reproductive axis. These experiments performed in both Syrian and Djungarian hamsters question the well established TSH-T3-RFRP/Kp pathway for the seasonal control of reproductive activity and point to a very early TSH-independent change in Dio3 expression in this process.

## Results

For each experimental activation procedure, three different sets of parameters were analyzed in Syrian and Djungarian male hamsters: peripheral reproductive parameters (FSH, testes weight and seminal vesicle weight, the latter being a marker of testosterone exposure in the preceding days and weeks); central neuropeptides regulating GnRH neuron activity (RFRP in the dorsal hypothalamus and Kp in the arcuate nucleus) and markers of the melatonin signal integration and local thyroid hormone metabolism (*TSHβ* in the *pars tuberalis*, *Dio2* in the β1 tanycytes, *Dio3* in the α tanycyte cell bodies and MCT8 in the α tanycyte cell bodies).

### Photoperiodic activation of the reproductive axis in the male Syrian hamsters

**Figure 1:**
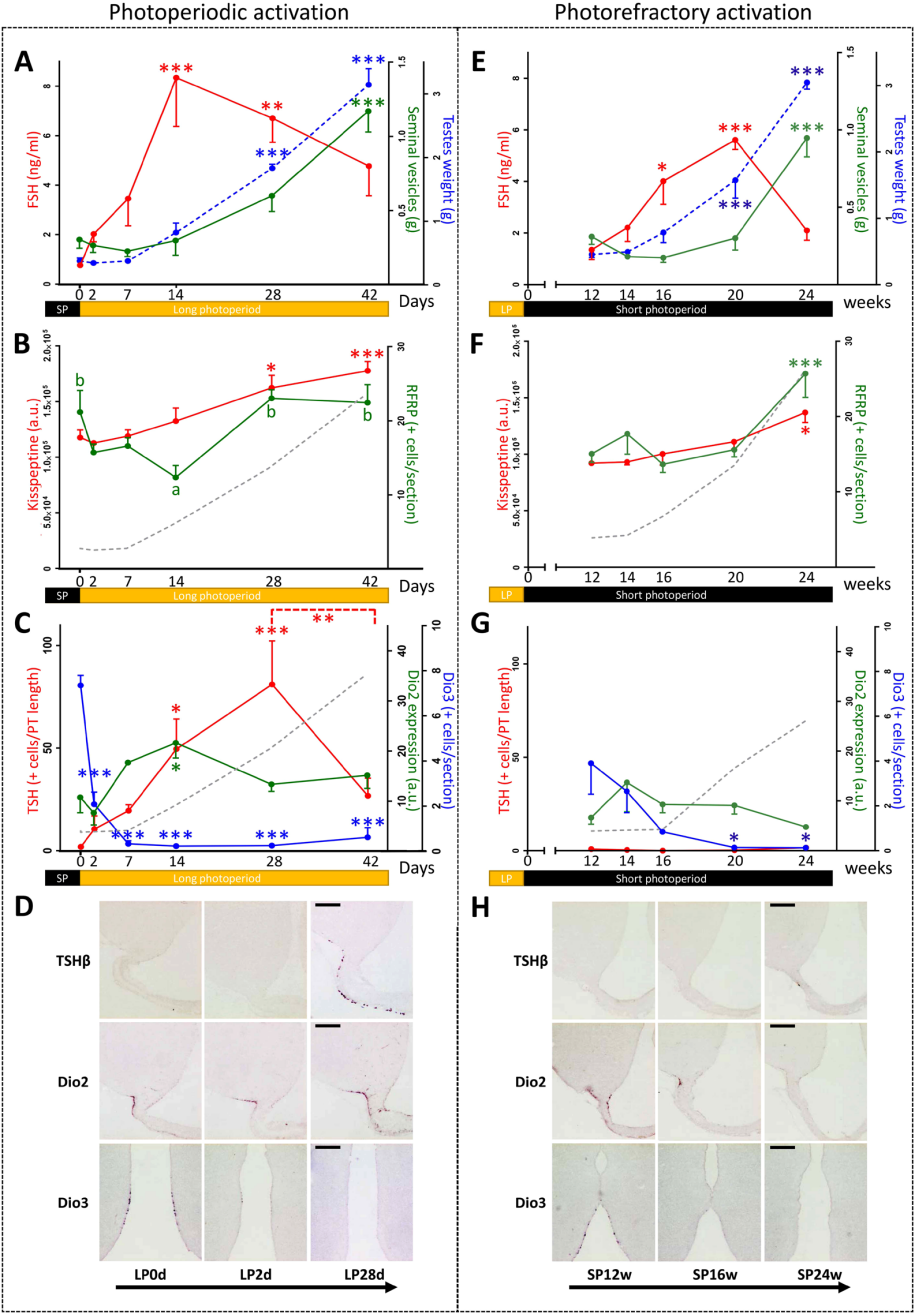
Photoperiodic **(A**–**D)** and photorefractory **(E**–**H)** activation of the reproduction in male Syrian hamsters: (**A** & **E**) time course of physiological parameters: combined testes weight (g) (blue dotted line), plasma FSH concentration (ng/ml) (red line), combined seminal vesicle weight (g) (green line). **(B & F)** Time course of neuropeptide expression: *Kiss1* integrated staining density of arcuate cell bodies (a.u.) analyzed by non-radioactive *in situ* hybridization (red line), number of *Rfrp* positive cells per section analyzed by non-radioactive *in situ* hybridization where different letters indicate significant differences (green line), combined testes weight (g) (grey dotted line). **(C & G)** Time course of the hypothalamic TSH/T3 axis: *Dio2* mean staining intensity (a.u.) analyzed by non-radioactive *in situ* hybridization (red line), *Dio3* positive alpha tanycytes per section analyzed by non-radioactive *in situ* hybridization (blue line), *pars tuberalis TSHβ* positive cells (green line) analyzed by non-radioactive *in situ* hybridization, combined testes weight (g) (grey dotted line). **(D & H)** Representative micrographs of the non-radioactive *in situ* hybridization stain for *TSHβ*, *Dio2* and *Dio3*: three time points are presented for each reactivation protocol: 0, 2d, 28d of long photoperiod (LP) for the photoperiodic activation; 12, 16, and 24w for the photorefractory reactivation. Scale bars = 200 µm.

Transfer of SP-adapted hamsters to LP conditions led to a progressive reactivation of the HPG axis (Fig. 1A–C). FSH plasma concentration reached a maximal value (8.34 ± 1.97 ng/ml) 14 days after the LP transfer and remained elevated up to 42 days thereafter. Following this increase in FSH, both testes and seminal vesicle weights became significantly higher 28 and 42 days respectively after the LP transfer. *Kiss1* expression measured as the mean integrated density of the hybridization signal per neuron increased after 28 and 42 days in LP. Notably, the number of *Rfrp* positive neurons initially decreased to a minimum value 14 days after the LP transfer and then increased to a maximal and stable value after 28 and 42 days in LP. The number of *TSHβ* positive cells in the *pars tuberalis* increased after 14 and 28 days in LP, and then dropped to lower levels at the 42nd day in LP (Fig. 1C,D). *Dio2* expression in β tanycyte end feet increased only at the 14th day in LP whereas *Dio3* expression in α tanycyte cell bodies displayed a pronounced and rapid decrease with significantly lower values reached already after 2 days in LP (Fig. 1C,D).

### Photorefractory reactivation of the reproductive axis in male Syrian hamsters

An endogenous (photorefractory) reactivation of the HPG axis was observed when hamsters were exposed to a prolonged SP (Fig. 1E–G). After 12 weeks of SP exposure, the HPG axis was fully inhibited as confirmed by minimum FSH levels and testes weights. Then after 16 weeks in SP, plasma FSH increased followed by a significant increase in testes weight after 20 weeks and then in seminal vesicle weight after 24 weeks in SP. An increase in both *Kiss1* and *Rfrp* expression was observed only after 24 weeks of SP exposure. Regarding the hypothalamic thyroid axis, *TSHβ* and *Dio2* expression remained low with no significant variation throughout the time course, while by contrast, *Dio3* expression started to decrease 16 weeks after SD exposure (Fig. 1G,H).

### Photoperiodic and photorefractory activation of the reproductive axis in male Djungarian hamsters

**Figure 2:**
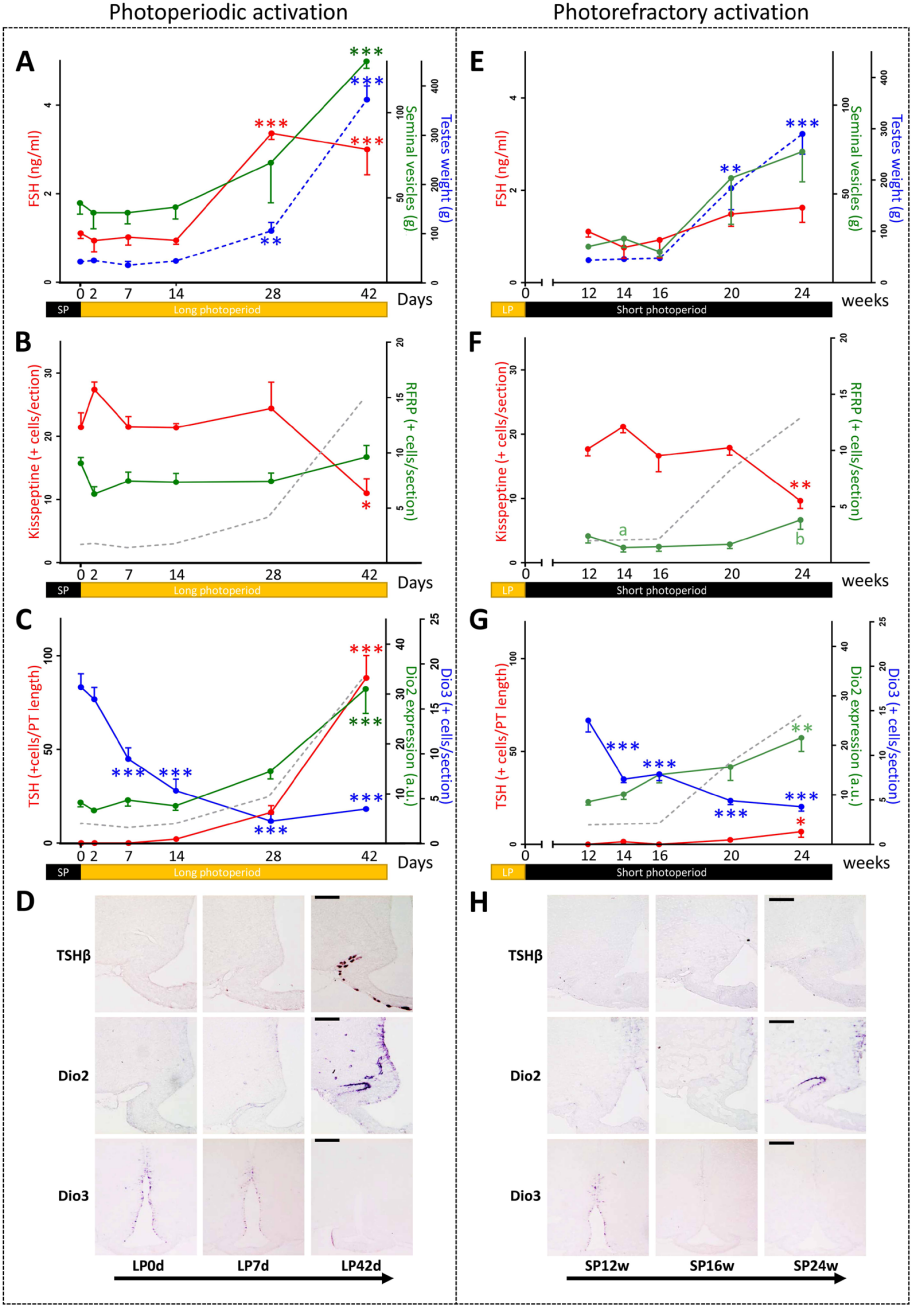
Photoperiodic **(A**–**D)** and photorefractory **(D**–**H)** activation of the reproduction in male Djungarian hamsters: **(A & E)** time course of physiological parameters: combined testes weight (g) (blue dotted line), plasma FSH concentration (ng/ml) (red line), combined seminal vesicle weight (g) (green line). **(B & F)** Time course of neuropeptide expression: number of arcuate KP immunopositive (JLV1 antiserum) neurons per section (red line), number of RFRP-3 immunopositive (GA197 antiserum) neurons per section where groups with different letters indicate significant differences (green line), combined testes weight (g) (grey dotted line). **(C & G)** Time course of the hypothalamic TSH/T3 axis: *Dio2* mean staining intensity (a.u.) analyzed by non-radioactive *in situ* hybridization (red line), *Dio3* mean staining intensity (a.u.) analyzed by non-radioactive *in situ* hybridization (blue line), *pars tuberalis TSHβ* positive cells (green line) analyzed by non-radioactive *in situ* hybridization, combined testes weight (g) (grey dotted line). **(D & H)** Representative micrographs of the *in situ* hybridization stain for *TSHβ*, *Dio2* and *Dio3*: three time points are presented for each reactivation protocol: 0, 2d, 28d of long photoperiod (LP) for the photoperiodic activation; 12, 16, and 24w for the photorefractory reactivation. Scale bars = 100 µm for TSHβ and Dio2 staining and 200 µm for Dio3 staining.

**Figure 3:**
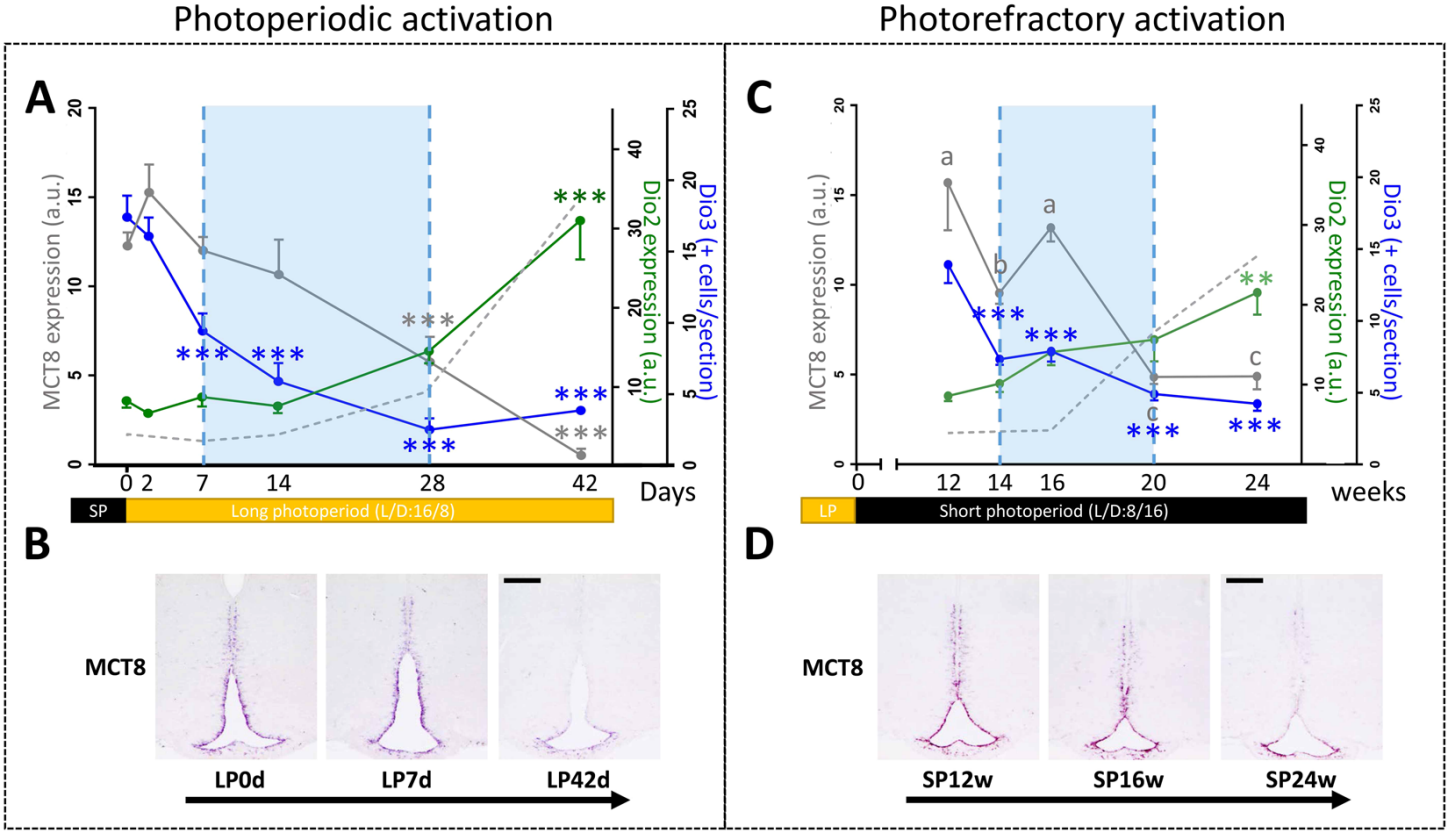
Analysis of the temporal expression of *MCT8*, *Dio2* and *Dio3* during photoperiodic **(A & B)** and photorefractory **(C & D)** activation of the reproduction in male Djungarian hamsters: **(A & C)** time course of hypothalamic *Dio2*, *Dio3* and *MCT8* expression: *Dio2* mean staining intensity (a.u.) analyzed by non-radioactive *in situ* hybridization (green line), *Dio3* mean staining intensity (a.u.) analyzed by non-radioactive *in situ* hybridization (blue line), *MCT8* mean staining intensity (a.u.) analyzed by non-radioactive *in situ* hybridization where different letters indicate significant differences (grey line), combined testes weight (g) (grey dotted line). The blue shading represents a potential time window of high T3 resulting from reduced *Dio3* expression combined with high *MCT8* expression. **(B & D)** Representative micrographs of the *in situ* hybridization stain for *MCT8* expression. Three time points are presented for each reactivation protocol: 0, 2d, 28d of long photoperiod (LP) for the photoperiodic activation; 12, 16, and 24w for the photorefractory reactivation. Scale bars 200 µm.

A similar protocol of time-dependent investigation of both the photoperiodic and photorefractory activation of the reproductive axis was performed in Djungarian hamsters to investigate putative species-dependent differences in these processes (Fig. 2). During photoperiodic reactivation, circulating FSH and testes weight started to increase 28 days after the LP transfer, followed by a significant rise in seminal vesicle weight observed after 42 days in LP (Fig. 2A). The number of Kp and RFRP neurons remained stable during the LP reactivation, except after 42 days in LP where KP was decreased (Fig. 2B). *Rfrp* expression should however increase after the end of the time course because a significant difference exists between the LP42d group and a control group constantly maintained in LP (Supplemental Fig. 1). *Dio3* expression decreased already 7 days after the LP transfer while the increase in both *TSHβ* and *Dio2* expression was significant only at the 42^nd^ day. In this species, we also investigated the dynamic changes in the gene encoding the tanycytic thyroid hormone transporter MCT8, which displayed a major decrease of its expression 28 and 42 days after the LP transfer (Fig. 3).

When Djungarian hamsters were maintained in SP for a prolonged period, an endogenous photorefractory increase in testes weight was observed after 20 weeks in SP. Although the mean seminal vesicle weight increased notably in parallel with testes weight, this increase failed to reach statistical significance due to high interindividual differences (Fig. 2E). After 24 weeks in SP, the number of KP neurons decreased, while that of RFRP neurons increased when compared to the minimum number observed at 14 weeks in SP (Fig. 2F). *Dio3* expression in the tanycytes was the earliest gene to show a significant decrease already after 14 weeks in SP (Fig. 2G,H) whereas *Dio2* and *TSHβ* expression displayed a correlated increase after 24 weeks in SP (Fig. 2G,H). Notably, the photorefractory increase in *TSHβ* expression (reached after 24 weeks in SP), although significant, is very small (SP24w = 6.8 ± 3.0) and represents only 4.3 ± 1.9% of the level observed in the LP control animals (LP = 155.6 ± 10.5). *MCT8* expression displayed a significant decrease at the 20th week (Fig. 3C and D).

## Discussion

The objective of this study was to integrate a dynamic temporal dimension in the study of the activation of reproductive activity in order to validate the current neuroendocrine model linking changes in photoperiod to changes in reproduction in seasonal species. Our results obtained in two seasonal rodents reveal a very early and conserved decrease in tanycytic *Dio3* expression before any significant changes are observed for any of the other investigated parameters, whether the reproductive activation is induced by a long photoperiod or by endogenous photorefraction.

The current model of photoperiod integration and activation of the vertebrate HPG axis was mainly built by comparing the two reproductive states adapted to either LP or SP^3,5,7,11,12,15,16^. These investigations have highlighted the LP-induced increase in *pars tuberalis TSHβ* and tanycyte *Dio2*, and the decrease in tanycyte *Dio3*, leading to an increase in hypothalamic T3. Experiments showing that exogenous central administration of TSH or T3 in SP-inhibited hamsters restored reproductive activity led to the hypothesis that this photoperiodic regulation of the intrahypothalamic thyroid hormone metabolism is pivotal for the control of seasonal reproduction^7,8,13^. Furthermore, our previous studies showed a marked melatonin-dependent photoperiodic regulation of the reproductive neuropeptides KP^11^ and RFRP^17^, together with their ability to restore a reproductive LP-like phenotype after central infusion in SP-adapted hamsters^10,11^. Altogether, this led to the hypothesis that TSH and thyroid hormones restore reproductive activity via a regulation of these hypothalamic neuropeptides^8,18^.

When SD-adapted Syrian and Djungarian hamsters were transferred to a stimulatory LP, a progressive correlated increase in *TSHβ* and *Dio2* is observed, which is in agreement with the current view that TSH secreted from the *pars tuberalis* acts on its receptors on tanycytes to increase Dio2 expression^5,19,20^. Melatonin has been reported to regulate TSHβ and Dio2 expression within a few days^21,22^. In sheep, a significant 2.5 fold increase of TSHβ is observed 3 days after the transfer from SP to LP suggesting a fast integration of the new photoperiod^23^. Yet, it is unknown what minimum increase in TSH synthesis is required to induce a significant increase in Dio2 activity. A striking observation when both hamster species were transferred to the stimulatory LP, was the very early and sharp decrease in *Dio3* gene expression occurring well before any significant changes in *TSHβ* or *Dio2* expression. This finding raises the question of the photoperiodic regulator of tanycytic *Dio3* expression. Although earlier work suggested a coordinated opposite TSH-driven regulation of *Dio2* and *Dio3* ^6,20,24,25^, our data clearly indicate that an early LP-induced TSH-independent mechanism might inhibit *Dio3* expression. Interestingly, a recent study analyzing the natural winter to summer activation in the Djungarian hamster reported a similar decrease in *Dio3* several weeks before the increase in *Dio2* ^26^. Therefore TSH cannot be a direct upstream regulator of Dio3 in the reproductive reactivation pathway. *In silico* analysis of the *Dio3* promoter reveals potential binding sites for retinoic acid receptors such as RAR or RXR. Retinoic acid might be a good candidate for a seasonal regulator of *Dio3* because previous studies already showed that CRBP2, a protein involved in retinoic acid signaling, is an early seasonally regulated gene^27^. In addition to TSH, other photoperiodically-regulated PT mediators can also be considered. Tanycytes express Neuromedin U receptors, but acute Neuromedin U injections into the cerebral ventricles do not modify Dio3 expression^24^. Chromogranin A displays a strong seasonal regulation in the sheep PT^28^ but its effects on tanycytes are so far unknown.

During photoperiod-induced activation of reproductive activity in the Syrian hamster, a significant increase in FSH secretion was observed after 14 days in LP (like *TSHβ/Dio2*) followed by the testes weight after 28 days and finally the seminal vesicle weight after 42 days (in agreement with^29^). In the Djungarian hamster, FSH secretion and testis weight were significantly increased after 28 days (2 weeks before *TSHβ/Dio2*) followed by an increase in seminal vesicle weight 42 days after transfer to LP (in agreement with^30^). These findings are in agreement with a previous study in Djungarian hamsters kept in natural conditions showing that testis weight increases before (end of February) TSH and Dio2 expression (end of April)^26^. Expression of the reproductive neuropeptides RFRP and KP was also increased only 2 weeks after FSH in Syrian hamsters and either unchanged (RFRP) or decreased (KP) 2 weeks after the FSH increase in Djungarian hamsters. In a previous study, we showed that a chronic intracerebroventricular TSH administration in SP-adapted Syrian and Djungarian was able to restore a long day pattern of KP and RFRP expression and sexual activity^8^. In the present study, the comparative kinetics of *TSHβ*, *KP/RFRP*, FSH and testis weight in both hamster species during transfer from inhibitory SP to stimulatory LP clearly suggest that other, faster, mechanisms must occur in addition to the TSH-driven increase in KP/RFRP stimulation of the reproductive axis^8,10,11,31^. Such mechanisms could involve a direct interaction between the tanycytes and the GnRH neuron terminals as already described during puberty onset and at the preovulatory LH surge^32–34^. Tanycyte processes cover GnRH nerve terminals at the level of the neurohemal junction and this glial coverage can modulate the reproductive status. This interaction could involve an effect of T3 via a rapid switch in the tanycytic *Dio2/Dio3* balance associated with a high expression of the thyroid hormone transporter MCT8 (see^35^ for review) or an effect of prostaglandin E2, as observed during the preovulatory LH surge^36^. Prostaglandin E2 produced by the tanycytes directly stimulates GnRH neurons via EP1 and EP2 receptors located on the GnRH terminals and cell body respectively^37,38^. The EP2 receptor is a Gs coupled receptor which increases the firing rate of GnRH neurons by a PKA dependent pathway^38^. The EP1 receptor is a Gq coupled receptor, increasing GnRH release through an increase of the intracellular calcium concentration at the GnRH terminal^38^. Further *in vitro* and *in vivo* pharmacological experiments will be required to disclose the mechanisms underlying this fast, neuropeptide-independent, seasonal activation of gonadotropin secretion.

Seasonal species kept in constant environmental conditions may display spontaneous changes in reproductive activity involving two different mechanisms. Circannual species, like sheep and the European hamster (*Cricetus cricetus*), show sustained cycles of reproductive activation and inactivation, whereas SP photorefractory species like Syrian and Djungarian hamsters show a spontaneous and stable reactivation of their gonadal activity after several weeks in constant SP (see^39,40^ for review). The cellular mechanisms driving these long term spontaneous changes in reproductive activity are still unknown.

As for the photoperiodic activation of the gonadotropic axis, this endogenous photorefractory activation appears to be triggered by an early decrease of tanycyte *Dio3* expression. As expected, the spontaneous increase in testes and seminal vesicle weight occurred between 20–24 weeks in both species^29,30^. This gonadal activation was preceded by an increase in circulating FSH only in the Syrian hamster. Surprisingly and contrary to a previous study^41^, we did not observe such an increase of FSH in the Djungarian hamster. Even more surprisingly, as for the photoperiodic activation, RFRP and KP both responded only about 4 weeks after the increase in testes weight in both species. Furthermore, *TSHβ* and *Dio2* remained at constant low levels in the Syrian hamster, and displayed only a slight and late increase in the Djungarian hamster.

Altogether these observations support the hypothesis that the spontaneous photorefractory reactivation of the reproductive axis is independent of TSH production by the *pars tuberalis* and Dio2 activity in the tanycytes. Again, the reduction in *Dio3* expression appears to be the trigger for this photorefractory activation. This observation is in clear contrast with the mechanisms involved in circannual rhythms, where significant changes in *TSHβ* and *Dio2* were observed in correlation with the reproductive status of the European hamster kept in constant conditions^42^ or the sheep placed in photorefractory LP or SP conditions^43^.

Interestingly, the LP-induced or photorefractory-driven decrease in *Dio3* is initially associated with a stable high level of MCT8. Therefore, the inhibition of the thyroid hormone catabolizing enzyme *Dio3* associated with high MCT8-driven thyroid hormone intake may be sufficient to create a functionally significant peak of T3 during a time window occurring just before the activation of gonadotropin secretion and gonadal activation (blue shading in Fig. 3). T3 administration to SP-inhibited hamsters was shown to be sufficient to restore an active gonadotropic axis^7,13^ but so far the cellular targets of T3 for the seasonal control of reproduction have remained unidentified. The reproductive neuropeptide RFRP and KP have been proposed to be regulated by hypothalamic T3^8,24^. However, our temporal analysis of LP-induced and photorefractory reactivation of the HPG axis shows that FSH secretion is increased several weeks before detectable changes in *Rfrp* and *Kiss1* expression. Thus we have to look for new potential targets of thyroid hormones for the early stages of the reactivation of the gonadotropic axis, maybe the tanycytes themselves. Indeed, a recent study showed that T3 appears to regulate genes such as Tmem252, Cndp1,Myl2 and Elovl3 in tanycytes in an autocrine/paracrine manner^44^.

The comparative analysis of the dynamic LP-induced and spontaneous SP refractory reactivation of the reproductive axis may help to better understand the underlying neuroendocrine mechanisms involved in both phenomena, especially because these mechanisms appear conserved in the two hamster species. One striking observation is the early decrease in tanycytic *Dio3*, independent of measurable changes in *pars tuberalis TSHβ* expression in both LP-induced and photorefractory activation of the HPG axis. This finding indicates that *Dio3* expression can be regulated by TSH-independent mechanisms. In LP stimulated hamsters, this decrease could be driven by a melatonin independent effect of light, but in photorefractory hamsters other mechanisms either intrinsic to the tanycytes or in upstream structures must trigger this decrease. As previously suggested, retinoic acid signaling appears as a candidate for this regulation. Alternatively, epigenetic methylation of DNA is also known to regulate Dio3 during the seasonal cycle^45^. Finally, miRNAs such as miR-155 and miR-200 which regulate GnRH directly as well as its transcriptional regulators during puberty might also be involved in this seasonal activation of the gonadotropic axis^46^.

## Conclusion

Our dynamic approach including temporal information challenges the current hypothetic pathway responsible for the photoperiod dependent and the photorefractory reactivation of the reproduction. We suggest the existence of 2 pathways for the reactivation of reproduction. A first pathway is Dio3 dependent and might trigger HPG reactivation via a thyroid hormone spike. The second pathway is TSH, Dio2 and neuropeptide dependent. This pathway is triggered after the first one to sustain and stabilize the reactivation. Future studies will have to identify the photoperiodic trigger for changes in Dio3, as our study shows that the previously identified trigger for Dio2 changes, the *pars tuberalis* produced TSH, clearly cannot be the trigger for changes in Dio3 expression.

## Material and Methods

### Animals

Animal experimentation was conducted in accordance with the French National Law implementing the European Communities Council Directive 2010/63/EU and the French Directive 2013-118. All animal procedures were reviewed and validated by the Animal Welfare Committee of the Animal Resource and Experimentation platform (Chronobiotron UMS 3415) of the Strasbourg Institute of Neuroscience. All efforts were made to minimize the number of animals used. Male Syrian hamsters (*Mesocricetus auratus*) and Djungarian hamsters (*Phodopus sungorus*) were bred and raised in our animal facilities. They were housed in a controlled photoperiod with constant temperature (20 °C +/− 2 °C), hygrometry (50% +/− 5%) and food and water *ad libitum*. After birth, Syrian hamsters were maintained in a long photoperiod (LP) of 14 hours of light and 10 hours of dark and Djungarian hamsters in 16 hours of light and 8 hours of dark to maintain a sexually active status. Animals started the experimental protocol after puberty and were between 3 and 12 months old at the day of the sacrifice.

For the photoperiodic investigation, 36 adult LP adapted Syrian and Djungarian hamsters were transferred to a short photoperiod (SP) (respectively 10 hours of light and 14 hours of dark or 8 hours of light and 16 hours of dark) for 10 weeks to induce a full inhibition of their reproductive axis. Then, they were switched back to LP and were sacrificed (6 animals per group) after 0, 2, 7, 14, 28 or 42 days of LP exposure.

For the photorefractory investigation, 30 adult LP adapted Syrian and Djungarian hamsters were transferred to SP and were sampled (6 animals per group) after 12 (SP12w), 14 (SP14w), 16 (SP16w), 20 (SP20w) or 24 (SP24w) weeks.

### Tissue sampling

Animals were euthanized during the early light phase (ZT6 to ZT8) by CO_2_ inhalation. Blood was sampled by intracardiac puncture. The hamsters were fixed by transcardiac perfusion with periodate-lysine-paraformaldehyde fixative. After fixation, the seminal vesicles were cut at their very basis and weighed immediately while taking care to avoid loosing seminal fluid. Both testes were dissected out and weighed. Brains were post fixed by immersion in fixative solution during 12 h, dehydrated and then embedded in polyethylene glycol^47^. Serial 10 µm thick sections were cut from the beginning of the *pars tuberalis* to the end of the mammillary recess with a microtome, mounted on SuperFrost® Ultra Plus slides (Thermo Scientific, MA, USA), dried for 15 min at 60 °C and stored at −80 °C until analysis.

### Non radioactive *in situ* hybridization

Riboprobes for genes encoding TSHβ, Dio2, Dio3, RFRP, Kiss1 and MCT8 (SLC16A2) (Supplemental Table 1) were transcribed from linearized plasmids in the presence of digoxigenin-labeled nucleotides according to standard procedures (DIG RNA labeling mix, Roche ref 11 277 073 910; DIG RNA Labeling Kit SP6/T7, Roche ref 11 175 025 910; Meylan, France). The *in situ* hybridization was performed as previously reported^48^. Briefly, polyethylene glycol sections were postfixed, digested for 30 min at 37 °C with 0.5 µg/ml Proteinase K (Roche Ref 03 115 828 001, Meylan, France) and acetylated twice for 10 min in 100 mM triethanolamine and 0.25% acetic anhydride. Hybridization was performed for 40 h at 60 °C with 200 ng/ml labeled probes in 50% formamide, 5x SSC, 5x Denhardt’s solution and 1 mg/ml salmon sperm DNA. Stringency rinses were performed for 6 × 10 min in 0.1x SSC at 72 °C. Digoxigenin labeled probes were detected with alkaline phosphatase labeled anti-digoxigenin antibodies (Roche ref 11 093 274 910, Meylan, France). Alkaline phosphatase activity was detected with bromochloroindolyl phosphate and nitroblue tetrazolium. Section were covered with CC/Mount™ (Sigma Aldrich ref C9368, MO, USA) and then coverslipped with Eukitt® (Sigma-Aldrich ref 03989, MO, USA).

### Immunohistochemistry

In intact Djungarian hamsters, *Kiss1* mRNA levels in the arcuate nucleus are particularly low, especially in long photoperiod due to a strong negative sex steroid feedback^49^. This makes quantitative analysis of *Kiss1* mRNA is difficult and unreliable. Therefore, we measured KP expression by immunohistochemistry which provides a stronger signal easier to quantify. For consistency in peptide analysis, RFRP expression was also analyzed by immunohistochemistry in this species.

KP immunoreactive neurons were visualized as previously described by an avidin-biotin detection system using a primary antiserum raised against full-length rat kisspeptin-52 (JLV-1)^50^ diluted 1/1,500. JLV1 shows no cross-reactivity with other members of the RFamide family of peptides^51^. Djungarian hamster kisspeptin peptide sequence is identical to that of the mouse, and the specificity of JLV1 in the Djungarian hamster has previously been described^49^. Bound KP antibodies were detected by a 60 min incubation with biotinylated donkey anti-rabbit secondary antibody (1/2,000; Jackson ImmunoResearch ref 711-065-152, PA, USA), followed by a 60 min incubation with Neutravidin HRP (Thermo Scientific ref 31030, MA, USA). Sections were developed for 30 min in 0,5 mg/ml Diaminobenzidine (DAB; Sigma-Aldrich ref D7304, MO, USA) and 0.003% H_2_O_2_ in Tris-Imidazole buffer (pH 7.5), resulting in a brown precipitate. Sections were dehydrated and then coverslipped with Eukitt® (Sigma-Aldrich ref 03989, MO, USA). RFRP-3 neurons were visualized with a rabbit anti RFRP-3 antiserum (antiserum GA197, kindly provided by G. Anderson^52^) diluted at 1/1,500. Djungarian hamster RFRP sequence shows only one Arginine to Serine substitution on the carboxyterminal amino acid of the immunogen sequence used by Rizwan *et al*.^52^ and the specificity of this antiserum for the Djungarian hamster has been previously described^49^. Primary antibodies were visualized with a donkey anti-rabbit secondary antibody coupled to AlexaFluor 594 (Invitrogen ref A-21207, CA, USA) diluted at 1/1,000. The sections were then coverslipped with 60% glycerol in phosphate buffered saline containing 0.02% Sodium Azide and 2.5% Diazabicyclooctane. (Sigma-Aldrich ref D2522, MO, USA).

### Image analysis and Statistics

For counts of the kisspeptin cells in the Arc, as well as RFRP cells, care was taken that the sampling included sections rostrally and caudally of these structures that did not contain labelled kisspeptin or RFRP cells in order to be certain that our sampling included the full extent of the structure of interest. For each gene/peptide analyzed, a series of 8 to 10 sections per animal, taken every 90 µm for the Djungarian hamster and every 130 µm for the Syrian hamster, was collected.

All slides of each experiment were processed at the same time to ensure identical treatment and development conditions for semi-quantitative image analysis. Maximum care was taken to avoid signal saturation during the development step. After *in situ* hybridization, micrographs were taken on a Leica DMRB microscope (Leica Microsystems, Rueil-Malmaison, France) with an Olympus DP50 digital camera (Olympus France, Rungis, France). For semi-quantitative evaluation of the *in situ* hybridization, all lighting parameters on the microscope and the camera software (Viewfinder Lite; Olympus) were standardized to ensure consistent stable lighting throughout the image capture procedure. All images for semi-quantitative staining intensity analysis were recorded as 256-level grayscale tagged image file format images. A background image of the slide without a section was taken for each slide. The images were then analyzed using the ImageJ software (W. S. Rasband, U.S. National Institutes of Health, Bethesda, MD, USA). The background image was subtracted from the corresponding sample image to compensate for inhomogeneities in the illumination of the image field.

Kisspeptin and RFRP analysis was done respectively in the arcuate nucleus and in the dorsal mediobasal hypothalamus. For *Kiss1 in situ* hybridization, the mean integrated density was measured with a fixed-size circle surrounding the labeled cells. Neurons were randomly selected in the arcuate nucleus provided the nucleus of the neuron was clearly visible. The mean integrated density was determined by measuring a minimum of 50 cells. Preliminary experiments showed that 50 cells are enough to provide a stable mean labeling intensity. For RFRP *in situ* hybridization, and for KP and RFRP immunohistochemical analysis, positive cells were counted only if the nucleus was clearly visible. The number of cells counted was then normalized by the number of sections analyzed located in the region of interest for the neuropeptides.

*Dio2* analysis was done on the tanycyte end feet in the tuberoinfundibular sulcus, while *Dio3* and *MCT8* were analyzed in the alpha tanycyte cell bodies. For Dio2 and MCT8 *in situ* hybridization we measured the background corrected mean pixel gray value in an area selected using the line tool of ImageJ software with the line width adapted to cover the cell body layer. In the Djungarian hamster, Dio3 *in situ* hybridization was quantified by the same method described above for Dio2 and MCT8. In Syrian hamsters however, the number of positive cells was low and positive tanycytes were well separated, thus we simply counted the stained cells along the mediobasal hypothalamus and normalized the counts by the number of sections analyzed.

TSHβ was analyzed in the *pars tuberalis*. We counted the number of *TSHβ* positive cells, which was then normalized by the length of *pars tuberalis* that was sampled.

For each analyzed gene or peptide, the final value shown in the figures is the mean of the individual values obtained for 5 to 6 animals. Although the initial number of animals was 6 per group, for some analyses one animal had to be excluded because the region of interest was not properly exploitable.

### FSH Assay

Plasma FSH levels were determined in a volume of 50 µl using an RIA kit, as described previously^10^.

### Statistical analyses

Results are shown as mean ± SEM (n = 5–6 per group). The data were analyzed by multiple group comparisons using one-way analysis of variance (ANOVA), followed by a Tukey’s Honestly Significant Difference post-hoc test, as appropriate. Statistical significance was set at Pvalue < 0.05.

## Electronic supplementary material

Supplemental Table 1 and Figure 1
