# Supplementary material for: Downregulation of Deiodinase 3 is the earliest event in photoperiodic and photorefractory activation of the gonadotropic axis in seasonal hamsters
Source: Sci Rep. 2017 Dec 18;7:17739. doi: 10.1038/s41598-017-17920-y (PMC5735130; doi:10.1038/s41598-017-17920-y)
Supplement: Supplementary file 1 — Supplemental Table 1 and Figure 1 [file 41598_2017_17920_MOESM1_ESM.doc]

**Downregulation of Deiodinase 3 is the earliest event in photoperiodic and photorefractory activation of the gonadotropic axis in seasonal hamsters**

MILESI Sebastien, SIMONNEAUX Valerie and KLOSEN Paul

Supplementary Information

**Supplemental Informations :**

**
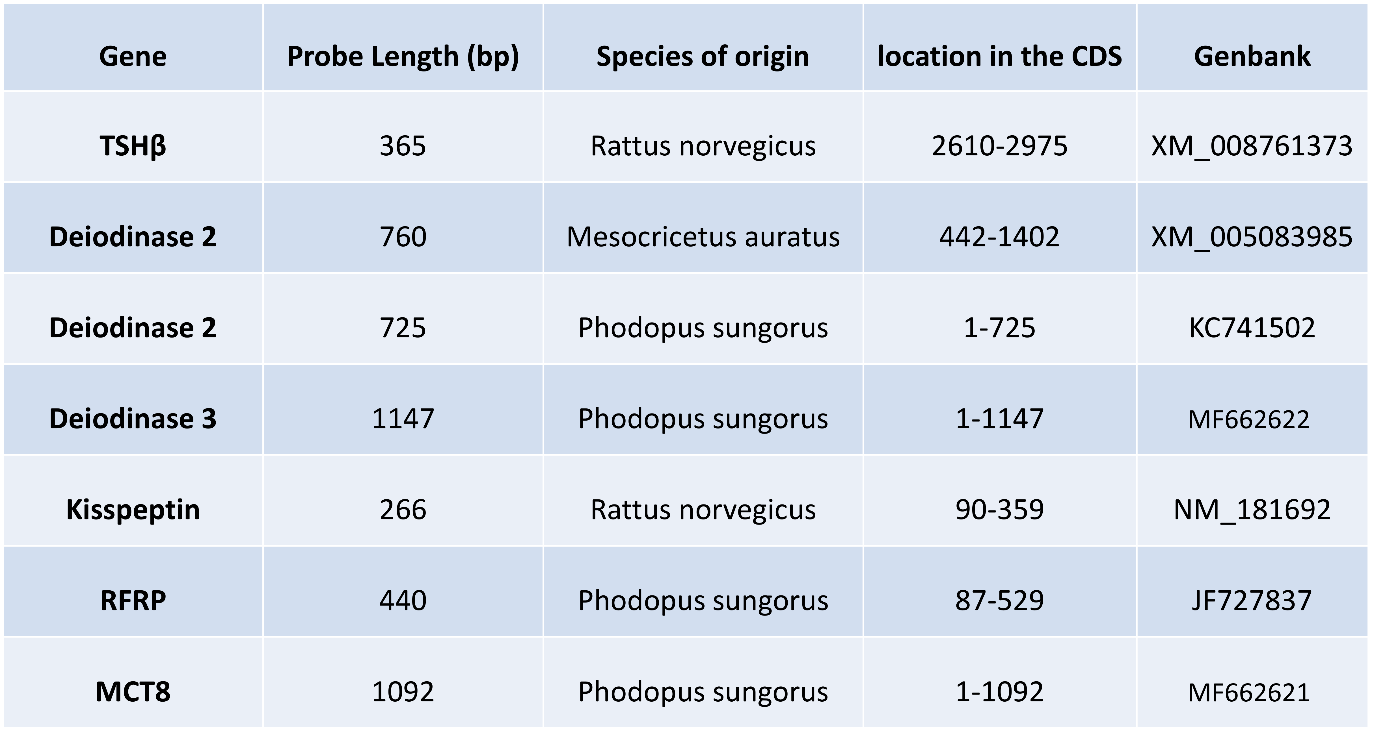
**

**Supplemental Table 1: *In situ* hybridization riboprobes**


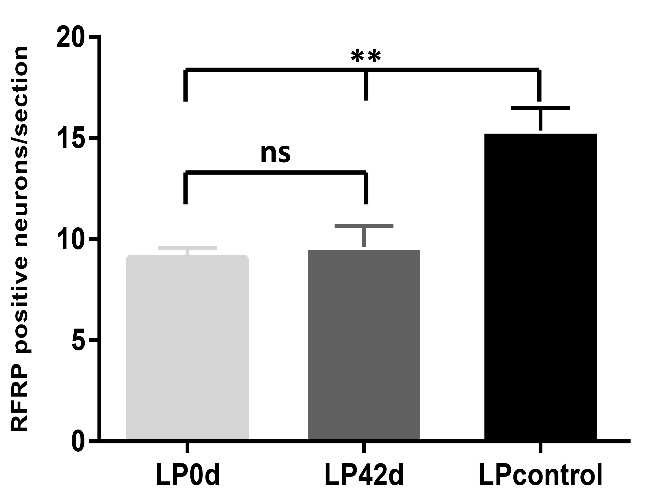


**Supplemental Figure 1: RFRP immunopositive cells (GA197 antiserum) per section in the Djungarian hamster:** LP0d and LP42 values were obtained from short photoperiod adapted animals (LP0d) and short photoperiod adapted animals switched back to long photoperiod for 42 days (LP0d). LP control group animals were raised in long photoperiod (16h light / 8h dark) since birth. Non-parametric t-test.
